# Patterns of Co-Occurring Gray Matter Concentration Loss across the Huntington Disease Prodrome

**DOI:** 10.3389/fneur.2016.00147

**Published:** 2016-09-21

**Authors:** Jennifer Ashley Ciarochi, Vince D. Calhoun, Spencer Lourens, Jeffrey D. Long, Hans J. Johnson, H. Jeremy Bockholt, Jingyu Liu, Sergey M. Plis, Jane S. Paulsen, Jessica A. Turner, The PREDICT-HD Investigators and Coordinators of the Huntington Study Group

**Affiliations:** ^1^Neuroscience Institute, Georgia State University, Atlanta, GA, USA; ^2^The Mind Research Network, Albuquerque, NM, USA; ^3^Department of Electrical and Computer Engineering, University of New Mexico, Albuquerque, NM, USA; ^4^Department of Psychiatry, University of Iowa, Iowa City, IA, USA; ^5^Department of Biostatistics, College of Public Health, University of Iowa, Iowa City, IA, USA; ^6^Department of Electrical and Computer Engineering, University of Iowa, Iowa City, IA, USA; ^7^Department of Neurology, University of Iowa, Iowa City, IA, USA; ^8^Department of Psychology, University of Iowa, Iowa City, IA, USA

**Keywords:** disease progression, gray matter concentration, humans, magnetic resonance imaging, movement disorders, multivariate methods, prodromal symptoms

## Abstract

Huntington disease (HD) is caused by an abnormally expanded cytosine–adenine–guanine (CAG) trinucleotide repeat in the *HTT* gene. Age and CAG-expansion number are related to age at diagnosis and can be used to index disease progression. However, observed onset-age variability suggests that other factors also modulate progression. Indexing prodromal (pre-diagnosis) progression may highlight therapeutic targets by isolating the earliest-affected factors. We present the largest prodromal HD application of the univariate method voxel-based morphometry (VBM) and the first application of the multivariate method source-based morphometry (SBM) to, respectively, compare gray matter concentration (GMC) and capture co-occurring GMC patterns in control and prodromal participants. Using structural MRI data from 1050 (831 prodromal, 219 control) participants, we characterize control-prodromal, whole-brain GMC differences at various prodromal stages. Our results provide evidence for (1) regional co-occurrence and differential patterns of decline across the prodrome, with parietal and occipital differences commonly co-occurring, and frontal and temporal differences being relatively independent from one another, (2) fronto-striatal circuits being among the earliest and most consistently affected in the prodrome, (3) delayed degradation in some movement-related regions, with increasing subcortical and occipital differences with later progression, (4) an overall superior-to-inferior gradient of GMC reduction in frontal, parietal, and temporal lobes, and (5) the appropriateness of SBM for studying the prodromal HD population and its enhanced sensitivity to early prodromal and regionally concurrent differences.

## Introduction

Huntington disease (HD) is a progressive, heritable condition most frequently associated with involuntary movements, and also characterized by impairments in executive functioning (1–3), recognition of negative facial expressions (4), and odorants (5), reward and punishment processing, and impulsivity (6). It is caused by an abnormally large cytosine–adenine–guanine (CAG) repeat expansion on the *HTT* gene. Age at motor diagnosis (7–9) and rate of disease development (10) are related to CAG-expansion and can be used to index the prodromal (pre-diagnosis) period; greater expansion numbers are associated with earlier onset and more rapid progression. Persons with fewer than 36 CAG-repeats are not at risk of HD; the range of 36–39 is not fully penetrant, as individuals can die of other natural causes. CAG-expansion is fully penetrant for 40 or more repeats, as individuals in this range will develop diagnosable symptoms if they reach a sufficient age (1). Despite progress in elucidating the mechanisms of HD, there is currently no cure. Identifying brain regions and functions impacted at the earliest pre-diagnosis stages is crucial for developing therapies to halt or reverse this tragic condition.

The current analyses use the PREDICT-HD dataset (11) to examine patterns of gray matter concentration (GMC) reduction in prodromal HD. PREDICT-HD is a multisite study containing possibly the largest collection of imaging data from individuals at various prodromal progression stages (9). We present the first application of multivariate source-based morphometry (SBM) and the largest application of univariate voxel-based morphometry (VBM), to a prodromal HD population, using a subset of 1050 PREDICT-HD participants to characterize GMC across the prodrome. VBM allows whole-brain, voxel-by-voxel concentration or volume comparisons (12) and has previously been applied to smaller prodromal HD populations [see Lambrecq et al. (13) meta-analysis for GMC and Dogan et al. (14) meta-analysis for GM volume]. Most of these studies did not stage the prodrome or included symptomatic HD patients, precluding identification of the earliest-affected factors.

Source-based morphometry, by contrast, is a multivariate approach that performs independent component analysis (ICA) on the same segmented structural images used for VBM, to examine co-occurring GMC patterns (15). Like VBM, SBM is beneficial for examining changes across the whole brain, rather than being limited to regions of interest. Unlike VBM, SBM is capable of determining the co-occurrence of gray matter differences in various areas of the brain. It is also more robust at localizing these changes (15, 16). SBM has been successfully applied to other clinical populations, including movement disorders such as multiple sclerosis (17) and Parkinson’s disease (18). It has also been used to study schizophrenia (16, 19–25) and Alzheimer’s disease (26), which share key features with HD, such as delayed onset, regional and cellular selectivity of atrophy, and cognitive abnormalities. Additionally, SBM may be more sensitive than previous methods to earlier and more widespread structural differences (15, 16, 21, 27). It also ameliorates the multiple comparisons problem by decreasing the number of maps being tested for group differences and can capture noise in the data as separate components that can be easily removed (16, 25). The ability to capture patterns of covariation enables exploration of the diffusivity or specificity of anatomical changes (17), extending interpretation beyond what regions are affected to identification of regions that are affected together.

We predict an overlap between significant regions in SBM and VBM results, with SBM revealing a greater number of affected regions, particularly in the early prodrome. We anticipate differences in these components not only between cases and controls but also among different prodromal levels. We expect the caudate to be among the most significantly affected regions, grouped in a component with functional and spatial neighbors, such as the putamen, thalamus, and cortex. Other significant components are expected to also include functionally related regions.

## Materials and Methods

### Participants

A total of *N* = 1292 (1016 prodromal and 276 control) participants were analyzed. Of these, 242 were excluded because of conversion to HD diagnosis (*N* = 82) or relatedness to another participant (*N* = 160). The remaining dataset included 1050 individuals (Table 1): 831 prodromal (521 females, mean age = 40.94, SD = 10.86, range 18.84–71.27; 310 males, mean age = 42.23, SD = 10.82, range 20.06–82.53) and 219 controls (137 females, mean age = 46.27, SD = 10.86, range 20.38–68.61; 82 males, mean age = 45.90, SD = 13.59, range 19.15–85.74).

**Table 1:** **Participant demographics**.

CAP group	*N* (male/female)	Mean age ± SD in years	Mean years of education ± SD	Mean CAG-repeat number ± SD
Control	219 (82/137)	46.1 ± 11.9^a^	14.8 ± 3.0^d^	20.1 ± 3.5
Low	216 (72/144)	34.6 ± 9.0^a–c^	14.1 ± 3.7	41.0 ± 1.9
Medium	284 (108/176)	41.5 ± 9.9^a–c^	14.6 + 2.9	42.1 ± 2.2
High	331 (130/201)	45.9 ± 10.5^b^	14.0 ± 3.5^d^	43.6 ± 9.4

*Participant CAP groups with mean age, years of education, and CAG-repeat number ± the SD*.

*^a^The mean age of the control group was significantly greater than that of the medium and low groups*.

*^b^The high group mean age was significantly greater than that of the medium and low groups*.

*^c^The medium group was significantly older than the low group*.

*^d^The control group had significantly more years of education than the high group*.

*Significant differences are not displayed for CAG-repeat number, as CAP group is based on CAG-repeat number, and is thus expected to significantly differ among prodromal groups*.

All PREDICT-HD participants provided written, informed consent and were treated in accordance with protocols approved by each participating institution’s internal review board. All participants underwent genotyping prior to study enrollment. Healthy controls had fewer than 36 *HTT* CAG-repeats, while prodromal individuals had more than 35. Exclusion criteria included manifestation of any other central nervous system condition or unstable medical or psychiatric disorders (1).

Prodromal progression level (low, medium, or high) for this study was indexed by a CAP score for each participant’s scanning session. CAP is a widely used measure for staging the prodrome and was developed based on an accelerated failure time model analysis with the entire PREDICT-HD database. CAP score factors age at first session and number of *HTT* CAG-repeats to assess prodromal disease burden [CAP = (age at first measurement) × (CAG − 33.6)]. For the present study, participants were classified into low, medium, and high CAP groups based on the algorithm described by Zhang et al. (28).

### Imaging Parameters

High resolution anatomical MR images were collected at 32 collection sites (53 unique scanners) using General Electric, Phillips, and Siemens scanners with field strengths of 1.5 T (Tesla) or 3 T, using a variety of acquisition parameters. T1 images at each site were obtained using three-dimensional (3D) T1-weighted inversion recovery turboflash (MP-RAGE) sequences. Also, 1.5 T scans were collected using General Electric and Siemens scanners. The Siemens protocol was constructed to be similar to the General Electric scan parameters: GRAPPA factor, 900 ms TI (inversion time), 2530 ms TR (relaxation time), 3.09 ms TE (excitation time), 256 mm × 256 mm field of view (FoV), 10° flip angle, 240 coronal slices with 1 mm slice thickness, 256 × 128 matrix with 1/4 phase FoV, 220 Hz/pixel receiver bandwidth. Protocol for 3 T scanners commonly involved a sagittal localizing series followed by acquisition of an axial 3D volumetric spoiled gradient recalled acquisition in steady state (GRASS) sequence, using the following scan parameters: ~1 mm × 1 mm× 1.5 mm voxel size, 18 ms TR, 3 ms TE, 24 cm FoV, 20° flip angle, 124 slices with 1.5 mm slice thickness, 0 mm gap, 256 × 192 matrix with 3/4 phase FoV, number of excitations (NEX) of two.

### Preprocessing

For each participant, the highest-quality T1 images from the earliest available scanning session were used for analyses. Images were aligned with the anterior commissure–posterior commissure (AC–PC) plane, resampled with 1 mm isotropic voxels to correct for inhomogeneity (29), and preprocessed using the SPM8 software package.^1^ All images were segmented into gray matter, white matter, and cerebrospinal fluid, unmodulated (to isolate GM concentration) and normalized to the same SPM8 Montreal Neurological Institute (MNI) template. Voxel intensities of normalized, segmented images represent relative voxel GMC. Image voxels were re-sliced to 2 mm × 2 mm × 2 mm. Gray matter images were smoothed to a full-width-half-maximum (FWHM) Gaussian kernel of 10 mm, based on recommendations for using high cluster-forming thresholds to reduce false positive rates in VBM studies (30). Processed images were 90 × 109 × 91 voxels in size.

### Source-Based Morphometry

Source-based morphometry, using the GIFT Matlab toolbox,^2^ was used to apply ICA to preprocessed (segmented, normalized, unmodulated, and smoothed) GMC images. The number of components from the 1050 images was estimated to be 23 using a minimum description length (MDL) criterion modified to account for correlated voxels (31). Each image was converted to a one-dimensional vector, yielding one participant-by-voxel data matrix. Using the infomax algorithm for spatial ICA, this matrix was decomposed into a mixing matrix, representing the relationship between 1050 participants and 23 components, and a source matrix, representing the relationship between the 23 components and the brain voxels. For the mixing matrix, rows are scores signifying how much each of the 23 components contributes to one participant’s data, while columns signify how one component contributes to each of the 1050 participants. The source matrix rows denote the degree to which one component contributes to brain voxels, while the columns are scores representing how a single voxel contributes to each of the 23 components (15). For each participant, the decomposition yields a loading coefficient for each component, with each component as a spatial map. ICASSO (32) was used to confirm component stability (20 iterations), and components were visually inspected to ensure that they primarily represented gray matter, rather than white matter or ventricles. As all 23 components were stable (with a minimum stability of above 0.90 and 21 components above 0.95), each participant had 23 loading coefficients.

MRI scanner site (53 sites total), gender, and CAP group were included as fixed factors, and years of education, age, and age^2^ were included as covariates. To examine the effects of CAP group on each SBM structural imaging component, a multivariate analysis of covariance (MANCOVA) using the General Linear Model (GLM) framework (with 23 dependent variables consisting of loading coefficients for each of the components) was executed in SPSS (33), followed by a Sidak *post hoc* test (34), thresholded at *p* ≤ 0.05, to examine specific CAP-group pairwise contrasts. The primary contrasts of interest were comparisons of the control group with each CAP group (low, medium, and high), as these contrasts represent differences between unaffected controls and different prodromal progression levels. Other pairwise contrasts (GMC in low > medium and medium > high) are included in Figure S1 in Supplementary Material.

### Voxel-Based Morphometry

Voxel-based morphometry was carried out using SPM8^3^ to compare local average GMC voxel-by-voxel across the entire brain. Input files were the same segmented, unmodulated, smoothed gray matter images used for SBM. To confirm proper registration of participant images to the SPM template, a correlation test was run to compare each image to the template. All resulting correlations were between 0.82 (only 5 images) and 0.96 (34 images). This is within the normal correlation range for SPM8, with 87% of images yielding correlations of 0.9 or higher. As an additional check, images with the lowest correlations were inspected using SBM’s checkreg. Analyses of CAP-group main effects and all pairwise group contrasts were carried out within the GLM framework, using the same covariates used for SBM (age, age^2^, gender, years of education, and scanning site). The main effects of group were determined with an *F*-test, and two-sided *t*-tests were used to compare pairwise group differences. Results were thresholded at a family-wise error (FWE) rate of *p* ≤ 0.05. Both positive and negative contrasts were investigated.

### Statistical Analysis

For the SBM analysis, variance equality was confirmed through Levene’s test for equality of variance for each SBM component, to address the assumption of homogeneity of variance among CAP groups. Potential outliers were examined *via* scatter plots of component loading coefficients versus CAP group. Each scanner site was investigated for the presence of outliers or unbalanced participant demographics, and no such biases were found (see Table S2 in Supplementary Material for each site’s participant data). Effects of gender and age (independent of disease burden) on GMC were accounted for *via* their inclusion as a fixed factor and covariate, respectively, in all analyses. To control for the influence of age (independent of disease burden) on GMC, age and age^2^ were also included as covariates in all analyses.

## Results

Participant demographics at baseline scan are presented in Table 1. ANOVA *t*-contrasts revealed that the control group was significantly older than the medium [*t*_501_ = 4.79, *p* < 0.0001] and low [*t*_433_ = 11.39, *p* < 0.0001] groups. The high group was also significantly older than the medium [*t*_613_ = 5.30, *p* < 0.0001] and low groups [*t*_498_ = 8.04, *p* < 0.0001], and the medium group was older than the low group [*t*_501_ = 4.79, *p* < 0.001]. The control group also had significantly more years of education than the high group [*t*_535_ = 2.61, *p* = 0.0092].

### Main Effect of CAP Group

**Figure 1:**
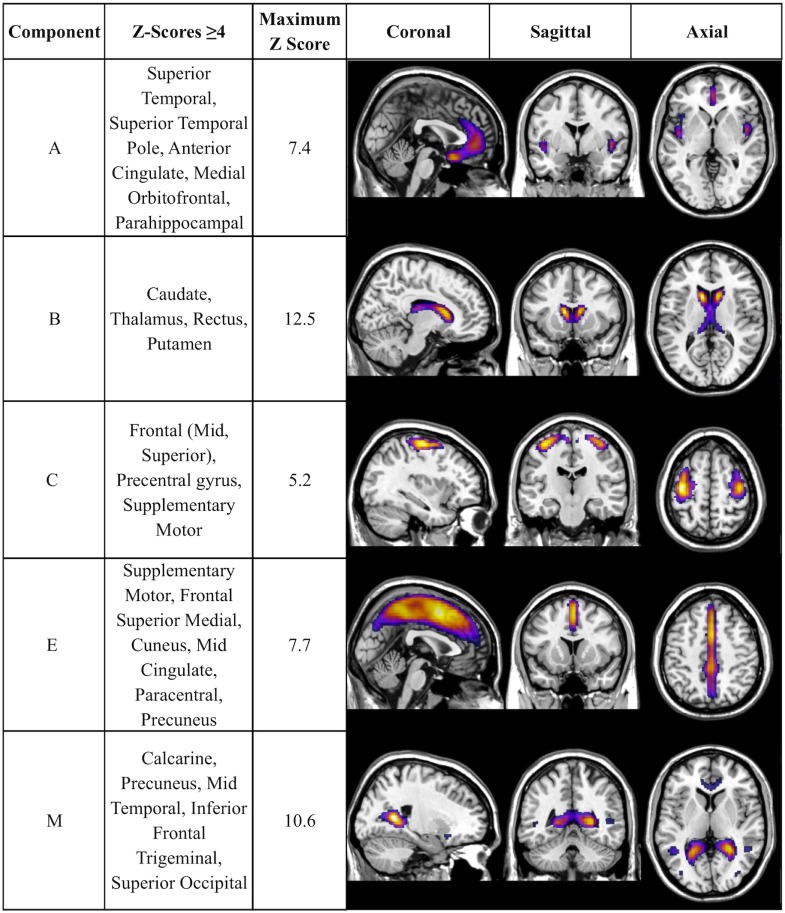
**Components with strongest CAP-group significance (*p* ≤ 0.0001)**. Multiview topography of Table 2 components, thresholded between *Z* = 4.0 and the component’s maximum *Z*-score (presented in column 3) to optimize display of regions with highest *Z*-scores.

**Figure 2:**
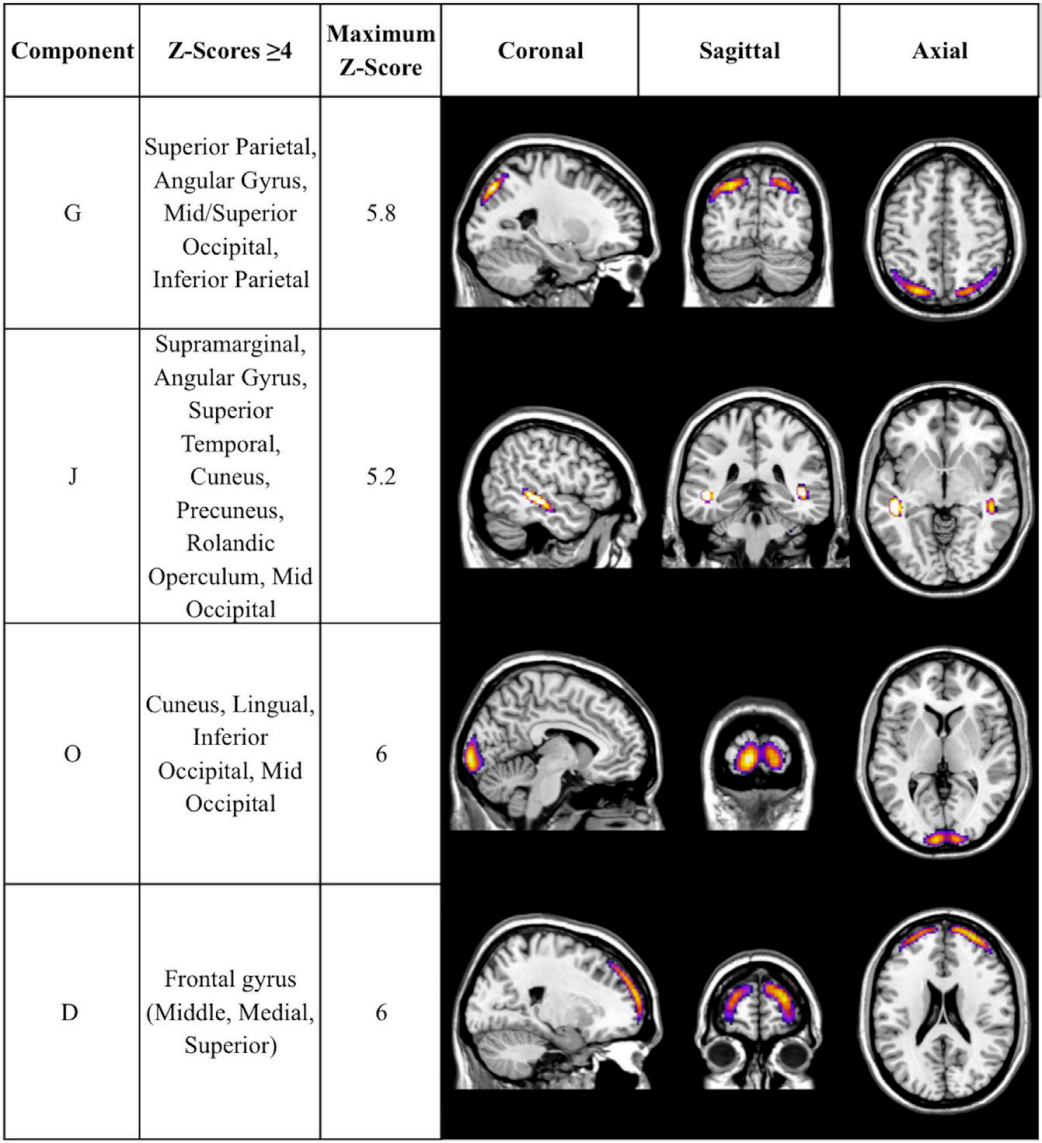
**Additional components with CAP-group significance of *p* ≤ 0.004**. Coronal, sagittal, and axial views of Table 3 components, thresholded to optimally view regions with maximum *Z*-scores. Regions displayed in each component have *Z*-scores between 4.0 and the component’s maximum *Z*-score, displayed in column 3.

**Figure 3:**
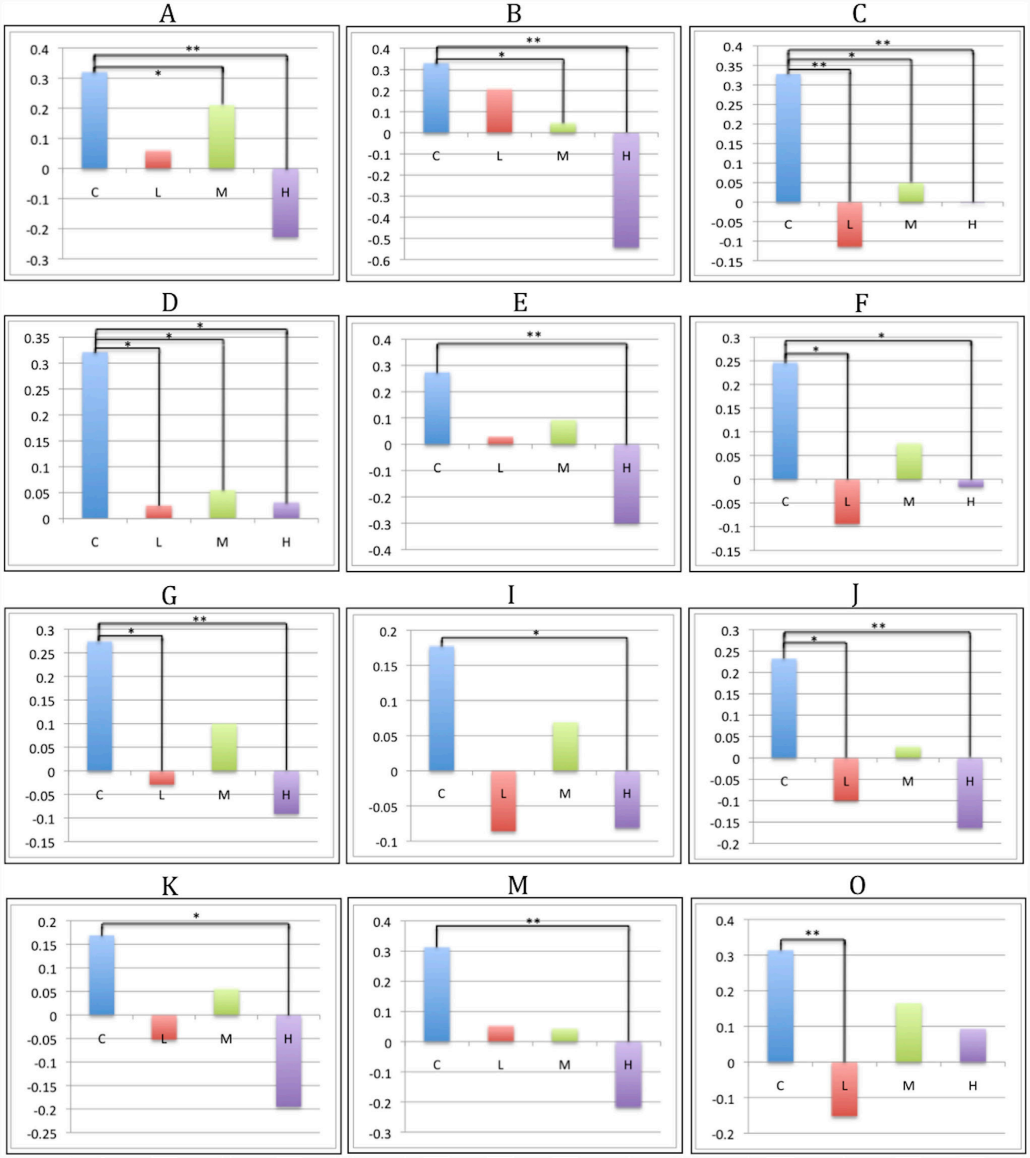
**CAP-group differences in SBM loading coefficients**. Significant pairwise group contrasts in CAP-group-significant components, derived from the Sidak *post hoc* test. Comparisons labeled with a single asterisk (*) denote significance of *p* ≤ 0.05; comparisons labeled with two asterisks (**) denote significance of *p* ≤ 0.0001.

There was a statistically significant omnibus effect of the mean weighted composite of SBM component loading coefficients for CAP group (*F*_69,1990.51_ = 3.92, *p* < 0.0001; Wilk’s Λ = 0.68, $\eta_{p}^{2}=0.12$). The MANCOVA revealed significant effects of CAP group on SBM loading coefficients from 12 of the 23 extracted components; the spatial maps are shown in Figures 1 and 2 and the primary brain regions involved in Tables 2 and 3. Each component’s loading coefficients displayed a progression gradient with means in the order of control > low > medium > high (Figure 3). The five components with the highest CAP-group significance [*p* < 0.0001; components A $\left(\eta_{p}^{2}=0.04\right)$, B $\left(\eta_{p}^{2}=0.092\right)$, C $\left(\eta_{p}^{2}=0.04\right)$, E $\left(\eta_{p}^{2}=0.04\right)$, and M $\left(\eta_{p}^{2}=0.04\right)$] contained neural sources spanning each lobe of the brain, in addition to the striatum, with the greatest contributions from frontal and temporal in addition to striatal areas. A complete list of components and encompassing regions is available in Table S1 in Supplementary Material.

**Table 2:** **SBM components with strongest CAP-group effects (*p* ≤ 0.0001)**.

Component	CAP effect statistic (*F*_3,688_)	Most significant regions	Left/right volume (cc)	Left/right maximum *Z* coordinates (*x*, *y*, *z*)
A	12.1915	Superior temporal pole	2.4/2.3	7.4 (−38, 13, −24)/7.2 (44, 16, −25)
		Superior temporal	2.8/2.2	5.8 (−49, −3, −3)/5.9 (51, 1, −8)
		Anterior cingulate/medial	1.7/2.2	5.1 (1, 44, 11)/5.7 (3, 50, −4)
		Frontal parahippocampal	0.3/0.2	3.9 (−14, −4, −23)/4.0 (18, 1, −24)
B	37.733	Caudate	3.8/3.9	12.5 (0.10, 17, 5)/11.6 (14, 20, 4)
		Thalamus	1.8/2.1	8.7 (−5, −13, 16)/8.3 (10, −15, 16)
C	11.948	Precentral/mid frontal	6.9/5.3	5.2 (−37, −6, 62)/4.9 (43, −4, 60)
		Precentral/superior frontal	4.2/2.7	4.6 (−34, −1, 55)/4.6 (30, −2, 63)
		Supplementary motor	1.1/1.0	4.2 (−5, −7, 66)/4.1 (13, −0, 66)
E	11.873	Supplementary motor	2.9/5.4	7.7 (2, 15, 49)/6.2 (6, 15, 47)
		Frontal superior	0.3/0.1	5.5 (0, 31, 33)/2.8 (2, −72, 31)
		Medial/cuneus	4.6/3.4	7.1 (2, −33, 60)/6.4 (6, −31, 58)
		Paracentral lobule	3.6/3.2	7.0 (2, −33, 60)/5.8 (6, 11, 46)
		Mid cingulate/precuneus	4.0/5.1	5.6 (2, −42, 56)/5.5 (6, −44, 62)
M	9.220	Calcarine	0.6/7.2	4.7 (−25, −60, 7)/10.6 (27, −50, 13)
		Left precuneus	1.9	6.9 (−25, −51, 14)
		Left mid temporal	1.0	4.5 (−46, −51, 12)
		Left superior occipital	0.5	4.1 (−20, −68, 20)

*Regions within components A, B, C, E, and M, including CAP-group effect *F* statistic derived from the Sidak *post hoc*, bilateral maximum *Z* scores with corresponding Montreal Neurological Institute (MNI) coordinates, and volumes in cubic centimeters (cc)*.

**Table 3:** **Additional SBM components with CAP significance of *p* ≤ 0.004**.

Component	CAP effect statistic (*F*_3,688_)	Most significant regions	Left/right volume (cc)	Left/right maximum *Z* coordinates (*x*, *y*, *z*)
G	5.650	Superior parietal	4.0/3.8	5.8 (−26, −71, 52)/5.0 (28, −70, 53)
		Angular gyrus	2.8/2.9	5.0 (−43, −65, 50)/4.6 (45, −59, 53)
		Left mid occipital	1.4	4.8 (−31, −80, 41)
		Left inferior parietal	0.4	4.3 (−35, −78, 41)
J	6.805	Right angular	1.2	5.2 (53, −48, 25)
		Superior temporal	7.7/7.1	4.9 (−58, −12, 1)/4.8 (51, −47, 21)
		Right precuneus	4.0	4.6 (14, −65, 24)
		Right superior temporal	2.9	4.6 (14, −65, 24)
		Right cuneus	1.2	4.4 (21, −65, 26)
O	9.418	Superior	6.5/6.5	8.1 (−7, −101, 9)/7.6 (17, −98, 3)
		Occipital/calcarine	0.9/1.1	6.5 (−12, −97, −3)/5.4 (32, −95, −0)
		Calcarine/inferior occipital	1.0/1.9	6.3 (−5, −97, 21)/6.0 (10, −95, 23)
		Occipital/calcarine	0.3/0.3	4.7 (−25, −96, 3)/5.9 (27, −93, 2)
D	3.88	Middle frontal	5.0/7.2	5.1 (−30, 57, 12)/6.0 (30, 54, 21)
		Superior frontal	8.3/11.3	5.1 (−30, 50, 25)/5.9 (30, 55, 17)
		Medial frontal	0.4/1.2	4.9 (−10, 65, 12)/5.1 (10, 65, 12)

*Regions within components G, J, O, and D (CAP-group significance of *p* ≤ 0.004), with CAP effect *F* statistics from the Sidak *post hoc*, bilateral maximum *Z* scores, and corresponding MNI coordinates and volumes*.

**Figure 4:**
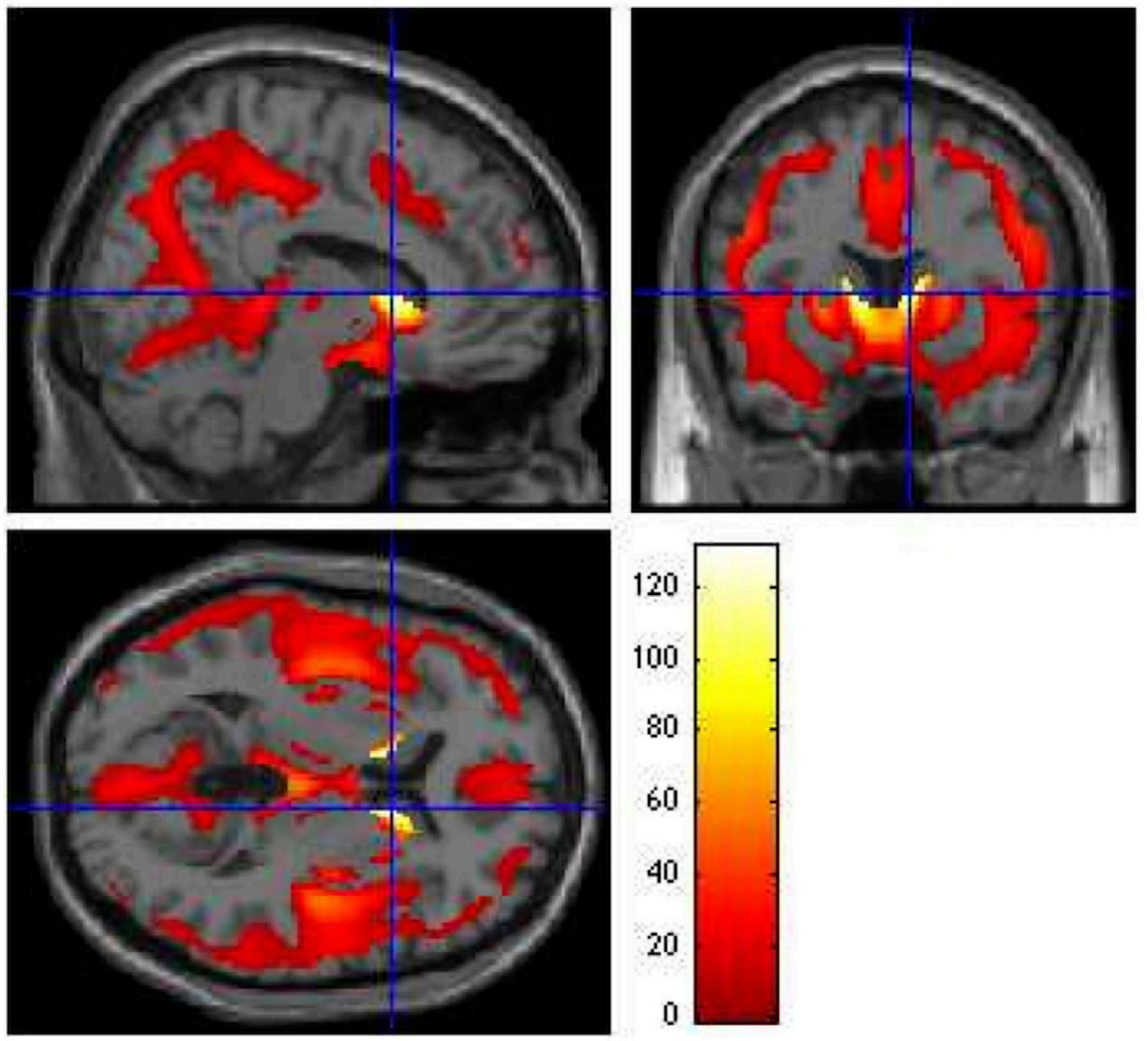
**Group main effects (ANCOVA *F* contrast)**. Main effects of CAP group on VBM, showing regions that vary throughout the prodrome (*p* ≤ 0.05). Global maximum: *x* = 10.00, *y* = 12.00, *z* = 6.00; *F*_3,992_ = 131.13. The colored bar legend codes the *F* statistic at each voxel, with white areas denoting voxels with the highest significance levels.

All VBM results were thresholded at a FWE rate of *p* ≤ 0.05. Similar to the SBM results, the voxel-based *F*-test within the GLM framework revealed widespread gray matter degradation throughout disease progression, with the strongest effects in the caudate [*F*_3,992_ = 124.18, *p* < 0.0001, *z* = Inf (left); *F*_3,992_ = 131.13, *p* < 0.0001, *z* = Inf (right)]. Other regions exhibiting overall group differences included inferior frontal orbital [*F*_3,992_ = 12.14, *p* = 0.001, *z* = 5.23 (left); *F*_3,992_ = 10.80, *p* = 0.008, *z* = 4.87 (right)], inferior occipital [*F*_3,992_ = 9.83, *p* = 0.028, *z* = 4.60 (left); *F*_3,992_ = 9.45, *p* = 0.044, *z* = 4.48 (right)], right inferior temporal (*F*_3,992_ = 9.36, *p* = 0.050, *z* = 4.45), hippocampus [*F*_3,992_ = 9.36, *p* = 0.049, *z* = 4.46 (left); *F*_3,992_ = 11.97, *p* = 0.002, *z* = 5.19 (right)], left lingual gyrus (*F*_3,992_ = 14.97, *p* < 0.0001, *z* = 5.93), and thalamus [*F*_3,992_ = 16, *p* < 0.0001, *z* = 6.16 (left); *F*_3,992_ = 13.71, *p* < 0.0001, *z* = 5.63 (right)] (Figure 4).

### Pairwise Group Contrasts

**Figure 5:**
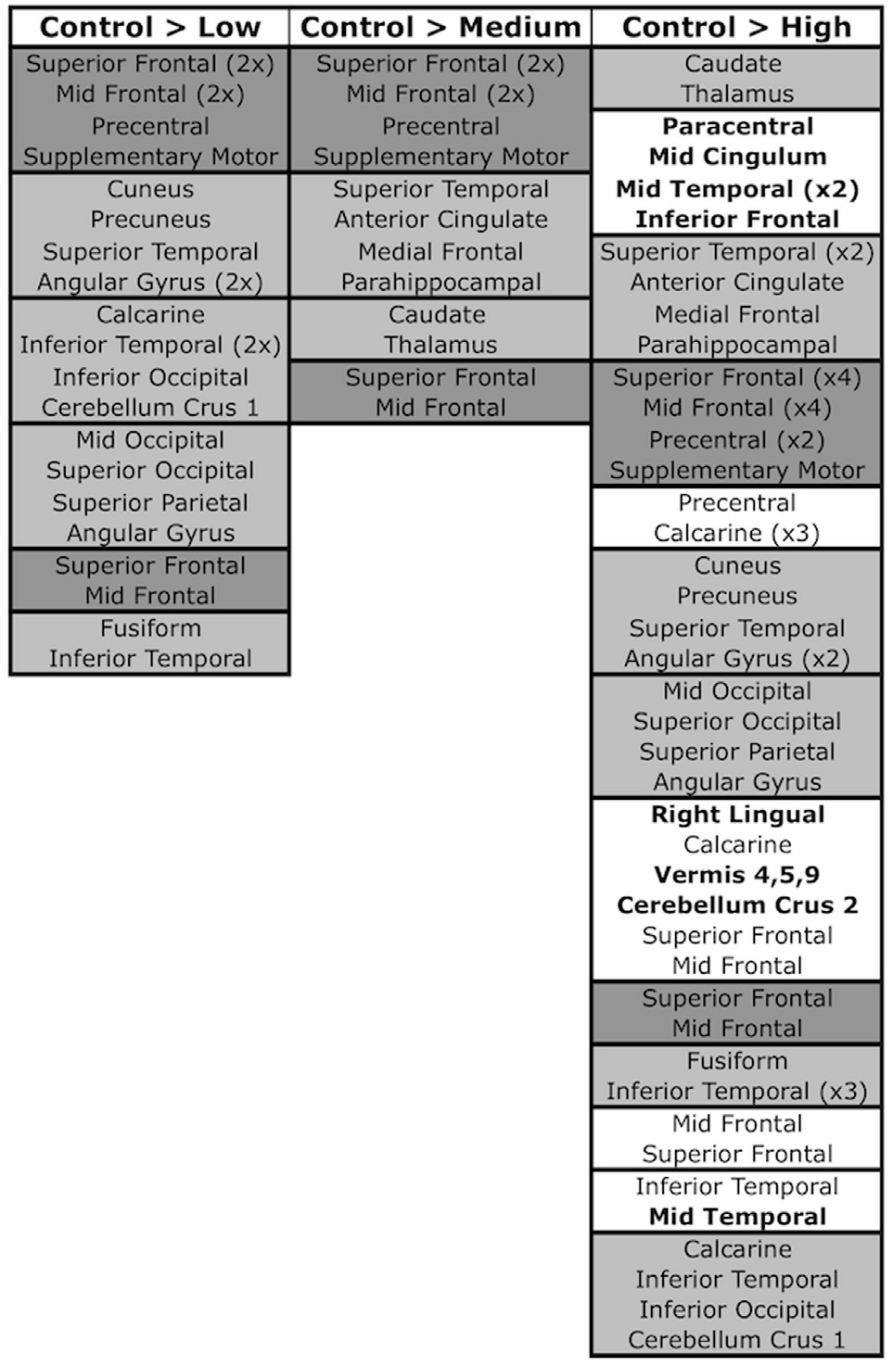
**SBM components and pairwise contrasts**. SBM regions significant in control > low, control > medium, and control > high contrasts. Pairwise contrasts are provided in Figure S1 in Supplementary Material. Regions are grouped into boxes, with each box representing a component that was significant in the contrast. Components are ordered by descending Sidak significance for the contrast (*p*-value). Regions within components are listed in descending order of their contribution to the component (*Z*-score). Dark gray boxes denote components/regions that were significant in all three contrasts, light gray boxes are components/regions that were significant in two contrasts, and white boxes (present only in the control > high contrast) contain components/regions that were significant in only the one contrast. Bolded regions within white boxes signify regions that did not appear in any other significant component/contrast.

The Sidak *post hoc* test yielded at least one significant pairwise CAP-group contrast for 13 of the 23 SBM components (Figures 3 and 5). In addition to capturing differences between control and prodromal groups, some components demonstrated differences between prodromal groups (such as low and medium groups). These components may most sensitively capture differences within the prodromal period (see Figure S1 in Supplementary Material for all supplemental contrasts). VBM pairwise group contrasts (Table 4) revealed the greatest group differences in contrasts with the high group. VBM pairwise contrast images are available in Figure S2 in Supplementary Material.

**Table 4:** ***T* statistics and coordinates (*x, y, z*) for regions significant in VBM pairwise contrasts**.

Region	Brodmann area	Cluster size	Peak *p*	Peak *T*	Talairach coordinates
**Control > medium contrast**					
Right caudate	25	268	<0.0001	5.83	(8, 10, 4)
Left putamen	48		0.005	4.98	(−6, 10, 2)
Right putamen	48	23	0.006	4.93	(22, 14, 4)
**Control > high contrast**					
Right caudate	25	26,289	<0.0001	15.51	(10, 12, 6)
Left caudate	25		<0.0001	14.51	(−8, 10, 4)
Left olfactory	–		<0.0001	11.86	(0, 8, 8)
Right cerebellum 6	–	303	<0.0001	5.8	(8, −74, 16)
Right mid temporal	21, 37	504	<0.0001	5.55	(56, −4, −18)
Right superior temporal	22		0.001	5.26	(−32, 36, −20)
Right inferior temporal	37	139	0.002	5.13	(48, −48, −24)
Right mid frontal	10, 46, 8	56	0.006	4.93	(−30, 56, 8)
Left inferior temporal	20	22	0.012	4.76	(−64, −44, −14)
Left mid temporal	22, 37	15	0.029	4.56	(−64, −26, −4)

*Significant regions in VBM pairwise contrasts. Significant regions with corresponding Brodmann areas, cluster sizes (in voxels), peak *p*-values, peak *T*-values, and Talairach coordinates are provided for VBM control > high-prodromal (near onset) and control > medium-prodromal contrasts. Values in front of parentheses are *T*_992_ scores; numbers inside parentheses are MNI coordinates (*x, y, z*)*.

### Low-Prodromal Group

The Sidak test revealed significant differences between the control and low groups in seven SBM components (O: *p* < 0.001, Cohen’s *d* = 6.2; C: *p* < 0.001, Cohen’s *d* = 7.1; J: *p* = 0.009, Cohen’s *d* = 4.6; G: *p* = 0.016, Cohen’s *d* = 4.3; F: *p* = 0.011, Cohen’s *d* = 4.5; D: *p* = 0.032, Cohen’s *d* = 4.1; H: *p* = 0.046, Cohen’s *d* = 3.9). These components contained contributions from middle and superior frontal gyrus (D and C), precentral gyrus and supplementary motor (C), occipital (O; G in mid/superior only; F in inferior only), calcarine (O and F), cuneus and precuneus (J), fusiform (H), superior (J) and inferior temporal (H and F), superior parietal (G), angular gyrus (G and J), and cerebellum crus 1 (F). By contrast, no substantial VBM differences were evident between control and low groups.

### Medium-Prodromal Group

Four SBM components were significantly different between the control and medium groups (C: *p* = 0.005, Cohen’s *d* = 4.8; A: *p* = 0.010, Cohen’s *d* = 4.5; B: *p* = 0.020, Cohen’s *d* = 4.2; D: *p* = 0.039, Cohen’s *d* = 3.9). These contained caudate and thalamus (B), frontal gyrus [superior (C and D), middle (A, C, D)], supplementary motor (C), precentral (C), superior temporal (A), anterior cingulate (A), and parahippocampal (A). For VBM, the medium group had significant reductions relative to controls, but only in right caudate (*T*_992_ = 5.83, *p* < 0.0001, Cohen’s *d* = 0.3) and putamen [*T*_992_ = 4.98, *p* = 0.005, Cohen’s *d* = 0.3 (left); *T*_992_ = 4.93, *p* = 0.006, Cohen’s *d* = 0.4 (right)].

### High-Prodromal Group

Thirteen SBM components were significantly different between the control and high groups [*p* < 0.0001: components A (Cohen’s *d* = 8.4), B (Cohen’s *d* = 13.1), C (Cohen’s *d* = 5.7), E (Cohen’s *d* = 7.8), J (Cohen’s *d* = 6.0), M (Cohen’s *d* = 7.2); *p* = 0.001: G (Cohen’s *d* = 5.5); *p* = 0.005: K (Cohen’s *d* = 4.7); *p* = 0.013: D (Cohen’s *d* = 4.3); *p* = 0.033: H (Cohen’s *d* = 3.9); *p* = 0.035: N (Cohen’s *d* = 3.9); *p* = 0.037: I (Cohen’s *d* = 3.8); *p* = 0.041: F (Cohen’s *d* = 3.8)]. Eight of these were significant in earlier control-prodromal contrasts (C > L, C > M). The remaining five components (E, I, K, M, and N) were not significant in earlier contrasts but contained regions within components that were significant in these contrasts, including putamen and thalamus (Q), mid- and superior-frontal (N), precuneus and calcarine (M), and inferior temporal (N, I). These components also contained regions not present in other contrasts, including paracentral (E), mid cingulum (E), mid temporal (M and I), inferior frontal (M), cerebellum 9 (K), right lingual (K), vermis 4, 5, 9 (K), cerebellum 9 (K), cerebellum crus 2 (K).

For VBM, the high group exhibited differences relative to controls in caudate [*T*_992_ = 14.51, *p* < 0.0001, Cohen’s *d* = 0.9 (left); *T*_992_ = 15.51, *p* < 0.0001, Cohen’s *d* = 1.0 (right)], right mid frontal (*T*_992_ = 4.93, *p* = 0.006, Cohen’s *d* = 0.3), right superior temporal (*T*_992_ = 5.24, *p* = 0.001, Cohen’s *d* = 0.3), right and left middle temporal [*T*_992_ = 5.55, *p* < 0.0001, Cohen’s *d* = 0.4 (right); *T*_992_ = 4.56, *p* = 0.029, Cohen’s *d* = 0.3 (left)], right and left inferior temporal [*T*_992_ = 5.13, *p* = 0.002, Cohen’s *d* = 0.3 (right); *T*_992_ = 4.76, *p* = 0.012, Cohen’s *d* = 0.3 (left)], right cerebellum (*T*_992_ = 5.80, *p* < 0.0001, Cohen’s *d* = 0.4), and left olfactory (*T*_992_ = 11.86, *p* < 0.0001, Cohen’s *d* = 0.8).

### Differences across the Prodrome

Components C (precentral gyrus, mid and superior frontal, supplementary motor) and D (middle and superior frontal gyrus) exhibited significant differences between the control group and each progression group (low, medium, high). Components G (superior parietal, angular, mid and superior occipital), H (inferior temporal, fusiform), J (angular gyrus, superior temporal, cuneus, precuneus), and F (calcarine, inferior temporal, inferior occipital, cerebellum crus 1) were similar but lacked significant differences between control and medium groups.

## Discussion

### Voxel-Based Morphometry

Our VBM GMC results coalesce well with existing literature. While many VBM studies have examined gray matter volume (GMV), one meta-analysis (13) compiled GMC VBM findings in prodromal and diagnosed HD. The GMC meta-analysis condensed 11 studies (297 patients and 205 controls) and reported a prodromal consensus on reductions in the left putamen and right inferior frontal gyrus (IFG) relative to controls. We replicated these findings in our PREDICT-HD sample and also found bilateral differences in caudate, thalamus, inferior frontal, hippocampus, and inferior occipital. Left lingual and right inferior temporal were also significantly affected.

According to the meta-analysis, symptomatic (but not prodromal) HD patients differed from controls in the left caudate body, left IFG, and right middle frontal gyrus, all of which were significant in our prodromal-control and prodromal level comparisons. This regional overlap is unsurprising; Although PREDICT-HD does not include HD cases, its incorporation of over twice as many participants as the meta-analysis lends increased statistical power. This explanation is bolstered by the particular prominence of these regional differences in our medium > high contrast, which suggests that these regions may be among the most dramatically affected in the late prodrome. Notably, the meta-analysis reported that HD (but not prodromal) patients consistently had reduced caudate GMC compared to control (and prodromal) subjects. The meta-analysis did not stage the prodrome, and the few studies it included that did index the prodrome had far fewer participants than the present study. In our large sample, we did not detect any VBM differences between the earliest prodromal group and the control group, and this may highlight the heterogeneity of the prodromal population. If a study considers the prodrome as a whole, yet has a disproportionate representation of early or late prodromal cases, certain differences may fail to replicate in many studies. This may be further compounded by methodological and parameter differences among studies, as well as individual participant trait differences across sites.

Our results align with those reported in the meta-analysis; however, its authors conclude that the data support a left-hemispheric degradation bias that extends bilaterally at the symptomatic stage. For their data, this is evident in the striatum, as the putamen and caudate demonstrated only left-hemispheric consensus significance. However, it is less supported by their results in other regions; right IFG was significant for the prodromal < control contrast, while left IFG was significant only in the HD < control condition. This would appear to suggest initial right-hemispheric changes in certain regions, which is supported by our findings. We observed stronger left-hemisphere effects in earlier prodromal contrasts in inferior frontal, putamen, thalamus, and inferior occipital, and significant findings in olfactory and lingual were limited to the left hemisphere. However, we also observed differences that were restricted to the right hemisphere in mid frontal, superior temporal, and cerebellum, as well as right-hemispheric differences preceding those in left-hemispheric mid/inferior temporal, hippocampus, and caudate (although caudate effects were overall bilateral). These results appear to support a hemispheric effect with regional variability.

### Source-Based Morphometry

Our SBM analysis revealed patterns both complementary to and distinct from those reported in previous VBM studies. Many imaging phenotypes were most reduced in the high CAP group (closest to diagnosis) and least perturbed in the low group, consistent with a low-to-high prodromal disease gradient. Both SBM and VBM identified strong GMC reductions in the high-prodromal progression group. Differences between control and low groups were present in several SBM components but were absent from VBM results, consistent with previous VBM–SBM comparison studies in which results were comparable, but SBM yielded additional regions that were not uncovered with the univariate method (16, 21, 25). This could indicate, as others have suggested (15, 16, 21), that multivariate SBM is more sensitive to early clinical differences in certain populations than similar univariate methods, such as VBM.

As the first application of SBM to prodromal HD, our results constitute novel evidence for SBM’s enhanced sensitivity to early prodromal HD differences. This benefit may be afforded by the inter-subject-variability present in heterogeneous clinical populations, such as prodromal HD, where differential CAG-expansion number and age confer a range of phenotypic severity in the population. A similar explanation was offered by the authors of a first-episode schizophrenia study in which SBM, but not VBM, yielded significant differences from controls (21); this was attributed to the heterogeneity of first-episode schizophrenia patients and the noise-dependency of the VBM method, both of which could be exacerbated by methodological problems or preprocessing differences.

In addition to early sensitivity, SBM may detect more widespread concentration differences throughout the prodrome. The SBM results captured each region present in the VBM results, as well as several other regions that were not detected with VBM (including thalamus, superior frontal, frontal inferior trigeminal, precentral, superior/inferior parietal, parahippocampal, hippocampus, precuneus, occipital, calcarine, lingual, fusiform, cuneus, anterior/mid cingulate, supplementary motor, paracentral lobule, fusiform, and areas in the vermis and cerebellar lobule VI). Furthermore, each region within the SBM components that yielded the greatest control-low differences (such as frontal regions) was only significant in VBM contrasts involving more advanced progression groups. This may be explained by SBM’s ability to integrate covarying brain regions and capture network-level differences, which contrasts with VBM’s focus on individual voxels that more robustly captures differences in small regions with few voxels.

The SBM results also provide insight regarding regions that are changing together, covariation that is captured by SBM’s grouping of regions into the same component. In many cases, regions within components were spatial or functional neighbors, exemplified by the presence of both the caudate and thalamus in component B; these regions work in concert with the subthalamic nucleus and substantia nigra to subserve coordinated voluntary movement (35). Frontal regions, in particular, tended to be grouped within the same components. Temporal regions were also more likely to dominate components than parietal and occipital regions, which were frequently grouped together. This may suggest that frontal and temporal regions change more independently from each other, while parietal changes may more frequently impact occipital areas and *vice versa*. While the components do not represent networks *per se*, the concurrence of these regional differences may highlight network-level changes by pinpointing areas where differences tend to coincide in participants. In light of the comparability, overlap, and extension of the VBM results present in the SBM results, the remainder of the discussion will focus on SBM component pairwise comparisons.

### Differences across the Prodrome

Two components (C and D) were sensitive to differences from controls at all three prodromal stages. Interestingly, both of these were dominated by frontal regions (superior frontal, mid/medial frontal, precentral, and supplementary motor). These areas may begin to change early in prodromal development and continue to be affected throughout its course, potentially making them appealing targets for early and ongoing interventions.

### Early Prodromal Differences (Control > Low)

Many SBM components exhibited differences between the control and low-prodromal groups, indicating widespread GMC loss even at early prodromal stages. The most commonly significant regions in this contrast were superior frontal, mid frontal, angular gyrus, and inferior temporal (each in two components). Overall, the strongest early effects were frontal (Component C: superior/mid/medial frontal, precentral, supplementary motor; Component D: superior/medial frontal). These areas are functionally relevant to HD, with supplementary motor cortex being integral to movement planning and coordination, and the precentral gyrus containing primary motor cortex (M1). A recent study examining prodromal functional connectivity of M1 reported that increased CAG-expansion was associated with reduced M1 connectivity with postcentral gyrus (primary somatosensory cortex) and visual centers in the cuneus (36). This could be accompanied by or related to gray matter degradation in these regions, aligning with their presence in group-significant SBM components. Component O, which contained widespread occipital and calcarine, was also highly affected in the early prodrome, consistent with reports of complex visual integration deficits being particularly discriminatory between symptomatic and prodromal individuals (37). The next most strongly affected component (J) contained cuneus, precuneus, superior temporal, and angular gyrus, possibly reflecting an early disruption of the default-mode network (which includes precuneus, angular gyrus, and temporal areas) (38). This component also implicates striatal pathways; the cuneus is connected to the angular gyrus, which associates with superior frontal regions and the caudate *via* the occipitofrontal fasciculus (39, 40).

### Later Prodromal Differences

Source-based morphometry first detected significant caudate and thalamus differences in the control > medium contrast (component B). Aside from the caudate, dorsomedial and anteroventral thalamic regions were the strongest contributors to this component. These subdivisions project to prefrontal and temporal cortices, respectively (41), both of which were also significant in this contrast (Components A, C, D). This thalamo-cortical projection is important for motor control, sensory relay (42), and many aspects of executive functioning (43), congruent with reported executive functioning impairments that worsen with increased prodromal progression.

The control > high-prodromal contrast captured each component that was significant in earlier prodromal contrasts. Within the five components that were not significant until the control > high contrast, many represented brain regions were also present in significant components from earlier prodromal contrasts, including precentral, calcarine, mid/superior frontal, and inferior/mid temporal. By contrast, late prodromal differences in the absence of earlier differences occurred in paracentral, mid cingulum, mid temporal, inferior frontal, lingual, vermis (4, 5, 9), and cerebellum crus 2. This suggests that, while many areas are affected early in the prodrome and continue to change throughout it, other (notably subcortical) regions may be substantially affected only at later prodromal stages. Several of these regions are important for motor performance, which is subtly affected in the early prodrome but declines steeply close to diagnosis-onset (1). The paracentral lobule, for example, interacts with supplementary motor cortex and distal limbs for somatosensation and is among regions that demonstrate reduced activation during processing of emotional faces in HD patients. The cingulum presents another example; the anterior cingulate (Component A) demonstrated intermediate and late prodromal differences, while the mid cingulate (Component E) only showed late differences. Anterior cingulate is involved in emotional regulation and has been implicated in depression and apathy (44), which co-occur in HD (1, 45, 46). Mid cingulate, by contrast, innervates premotor and motor cortical areas (47) and may contribute to increasing motor deficits with later prodromal progression.

### Summary

Frontal components were sensitive to early, intermediate, and late prodromal stages, with differences beginning earliest and remaining strongest in superior and mid frontal areas. Frontal differences were first observed (control > low contrast) in precentral, supplementary motor, and mid and superior frontal regions that tended to be grouped in the same components. These may be among the most continuously affected regions in prodromal HD.

Inferior frontal trigeminal was the only affected inferior frontal region and exhibited differences only in the late prodromal contrast. A similar pattern was observed in inferior temporal, which was much more typical and significant in the late compared to early prodromal contrast. Superior temporal and angular gyrus also displayed robust early prodromal differences that continued into the mid and late prodrome, while inferior parietal was absent from the results entirely. Overall, this suggests a superior-to-inferior gradient of GM degradation across the prodrome, with the exception of the occipital lobe (both superior and inferior occipital were significant in early and late contrasts).

Many occipital and subcortical regions did not exhibit concentration differences until the late prodromal stage, including cerebellum crus 2, vermis 4, 5, 9, right lingual gyrus, and mid cingulum. Significant inferior frontal trigeminal, mid temporal, and paracentral lobule effects were also limited to this contrast. This delayed contribution of regions important for movement is in accordance with motor impairment as the primary instigator of diagnosis, as well as the observed non-linear prodromal pattern of motor functioning that culminates with sudden deterioration in the late prodrome (1).

The overlap between results from SBM and VBM (the latter of which has been applied in other prodromal HD studies) suggests that SBM is suitable for studying this population. Consistent with previous findings in other clinical populations (15, 16, 21), SBM appeared to detect concentration differences at earlier progression stages and in more regions than VBM. Furthermore, the grouping of regions into SBM components highlights covarying areas that may be good targets for investigating network-level changes.

### Limitations

There are a number of important factors to consider while interpreting these results. In any study involving a large number of participants with a rare condition, multiple scanning sites are typical, and thus a certain degree of inhomogeneity in data collection is inevitable. This study used data from several unique 1.5 or 3 T scanners. Every effort was made to control for possible confounds relating to multiple collection sites. Uniform protocols were established to ensure maximal homogeneity of data collection, and field strength was included as a fixed factor in all analyses. As described in the Section “Statistical Analysis,” collection sites were examined for outliers and unbalanced participant demographics (site demographics are provided in Table S2 in Supplementary Material). It is also worth noting that a study investigating scan parameter effects on SBM components from a multisite schizophrenia study found that site effects were successfully eradicated with both GLM (VBM) and SBM correction (48).

Given the dramatic striatal role in HD, the lack of significant differences between the control- and low-prodromal groups in the caudate/thalamus component was surprising. This component was strongly significant in every other pairwise contrast (including supplemental contrasts low > medium and medium > high), indicating that SBM robustly captured prodromal progression in these regions. Additionally, as exemplified by the VBM GMC meta-analysis, caudate differences are not always found in the earliest prodromal contrasts (although this does not seem to be the case in volumetric analyses). Concentration measures may capture subtler changes in underlying cellular mechanisms that are not yet reflected by structural volumes, which may also underlie other dissimilarities between GMV and GMC results. Nonetheless, we investigated possible explanations for this finding. Although the SBM components were examined for and did not appear to contain artifacts, prodromal individuals may have smaller intracranial volumes (ICV) than controls (49), and it is important to investigate the possibility that ventricular artifacts in some components could mask GMC reduction. Unsurprisingly, ICV was significantly correlated with 19 of the 23 SBM components. However, when ICV was included as an additional covariate in the SPSS MANCOVA analysis, it did not substantially change either the MANCOVA results or the Sidak *post hoc* results (7 components were still significant for control > low, 4 for control > medium, and 11 for control > high). ICV also did not alter the caudate component effects, which was of particular concern given the unexpected lack of control > low significance. It may also be possible for subtle head motions that are undetectable during data collection to influence results (50), an inevitable possibility in any study investigating movement disorders using motion-sensitive equipment. While it was not possible to address this with the current dataset (beyond behavioral data and motion-correcting preprocessing steps), we aim to investigate possible motion effects using measures of framewise displacement from resting state data, available for some participants.

Source-based morphometry components generally followed an expected pattern of control > low > medium > high. However, the finding that seven components were significant for the control > low contrast compared to four for the control > medium contrast was an unexpected, but not totally unprecedented finding. Cognitive and clinical findings in the medium-prodromal group have also been less consistent than in the other groups (9). Explanations for these differences have varied; some have suggested potential protective effects in the early prodrome, aligning with observations of compensatory increased task-based connectivity and activity during performance of certain tasks (51). Even pathological nuclear protein aggregation is suspected of having a protective role, as it does not correlate well with disease symptoms or onset (52). Inconsistent group results could also reflect an imperfect parsing of prodromal groups, highlighting an underlying goal of this study (pinpointing additional sources of variation beyond CAG-expansion and age). We attempted to control for important group differences (like age) that could skew results. Additionally, our group and others are working to develop “risk scores” that encapsulate other factors in onset variability. However, the present heterogeneity in the medium group may account for some unexpected findings.

## Conclusion and Future Directions

Our findings support the utility of SBM for investigating prodromal HD. The results reinforce the acknowledged widespread nature of neural degradation in HD, with reduced GMC being evident across the entire brain rather than isolated to the striatum. They also provide insight into patterns of atrophy and concurrently affected regions; frontal and temporal areas differed relatively independently compared to (often co-occuring) parietal and occipital differences. Additional evidence was uncovered for differential patterns of decline across the prodrome, with the earliest differences affecting more superior areas, intermediate differences increasing in middle regions, and later differences in more inferior, occipital, and subcortical structures.

Future research would benefit from exploiting the increased sensitivity of morphometric techniques like SBM to address questions regarding how subtle structural differences relate to cognitive, motor, and psychiatric functioning. The parsing technique inherent in SBM can also be used to investigate differences between prodromal white and gray matter changes. Furthermore, it is possible that differences in *HTT* and other genes in the HD signaling pathway (53) underlie some observed structural differences. Investigation of genotypes associated with structural phenotypes may elucidate markers of disease progression that are present earlier than detectable deficits on clinical batteries.

## Author Contributions

VC contributed to idea generation and results interpretation, assistance with SBM technique, and manuscript preparation. SL and JDL assisted with modeling and improving statistical analyses and manuscript preparation. HB and HJ assisted with manuscript preparation, data management, organization, and preprocessing. JL contributed to manuscript preparation, training with SBM technique and parameters, and selection of the final dataset. SP preprocessed the VBM and SBM data. JP contributed to idea generation, assisted with manuscript preparation and submission, and provided PREDICT-HD data expertise and access. JT contributed to idea generation and experimental design, and manuscript preparation and submission.

## Conflict of Interest Statement

The authors declare that the research was conducted in the absence of any commercial or financial relationships that could be construed as a potential conflict of interest.

## Footnotes

^1^http://www.fil.ion.ucl.ac.uk/spm/software/spm8

^2^http://mialab.mrn.org/software/gift

^3^http://www.fil.ion.ucl.ac.uk/spm/software/spm8
