# Supplementary material for: Patterns of Co-Occurring Gray Matter Concentration Loss across the Huntington Disease Prodrome
Source: Front Neurol. 2016 Sep 21;7:147. doi: 10.3389/fneur.2016.00147 (PMC5030293; doi:10.3389/fneur.2016.00147)
Supplement: Supplementary file 1 [file Data_Sheet_1.doc]

Supplementary Material

# Patterns of Co-Occurring Gray Matter Concentration Loss Across the Huntington Disease Prodrome

Jennifer Ciarochi, Vince D. Calhoun, Spencer Lourens, Jeffrey D. Long, Hans Johnson, H. Bockholt, Jingyu Liu, Sergey Plis, Jessica A. Turner, Jane S. Paulsen, and the PREDICT-HD Investigators and Coordinators of the Huntington Study Group

***Correspondence:** Corresponding Author: jane-paulsen@uiowa.edu

Supplementary Data

PREDICT-HD Investigators, Coordinators, Motor Raters, Cognitive Raters

Executive Committee

Principal Investigator Jane S. Paulsen, Jeffrey D. Long, Hans J. Johnson, Thomas Brashers-Krug, Phil Danzer, Amanda Miller, H. Jeremy Bockholt, and Kelsey Montross.

Scientific Consultants

Deborah Harrington (University of California, San Diego); Holly Westervelt (Rhode Island Hospital/Alpert Medical School of Brown University); Elizabeth Aylward (Seattle Children’s Research Institute); Stephen Rao (Cleveland Clinic); David J. Moser, Janet Williams, Nancy Downing, Vincent A. Magnotta, Hans J. Johnson, Thomas Brashers-Krug, Jatin Vaidya, Daniel O’Leary, and Eun Young Kim (University of Iowa).

Core Sections

Biostatistics: Jeffrey D. Long, Ji-In Kim, Spencer Lourens (University of Iowa); Ying Zhang and Wenjing Lu (University of Indiana).

Ethics: Cheryl Erwin (Texas Tech University Health Sciences Center); Thomas Brashers-Krug, Janet Williams (University of Iowa); and Martha Nance (University of Minnesota).

Biomedical Informatics: H. Jeremy Bockholt, Jason Evans, and Roland Zschiegner (University of Iowa).

PREDICT-HD Coinvestigators

Amanda Miller, LMSW (University of Iowa, Iowa City, Iowa, USA, site investigator);

Edmond Chiu, MD, and Samantha Loi, FRANZCP, MBBS, BMedSc (St. Vincent’s Hospital, The University of Melbourne, Kew, Victoria, Australia, site investigators);

Phyllis Chua, MD (The University of Melbourne, Royal Melbourne Hospital, Melbourne, Australia, site investigator);

Lynn Raymond, MD, PhD (University of British Columbia, Vancouver, British Columbia, Canada, site investigator);

Christopher A. Ros s, MD, PhD (Johns Hopkins University, Baltimore, Maryland, USA, site investigator);

William M. Mallonee, MD (Hereditary Neurological Disease Centre, Wichita, Kansas, USA, site investigator);

Ali Samii, MD (University of Washington and VA Puget Sound Health Care System, Seattle, Washington, USA, site investigator);

Randi Jones, PhD (Emory University School of Medicine, Atlanta, Georgia, USA, site investigator);

Roger A. Barker, BA, MBBS, MRCP (John van Geest Centre for Brain Repair, Cambridge, UK, site investigator);

Elizabeth McCusker, MD, and Clement Loy, MD (Westmead Hospital, Sydney, Australia, site investigators);

Michael Orth, MD, PhD, and Sigurd Süβmuth, MD (University of Ulm, Ulm, Germany, site investigators);

Kimberly Quaid, PhD (Indiana University School of Medicine, Indianapolis, IN, USA, site investigator);

Mark Guttman, MD (Centre for Addiction and Mental Health, University of Toronto, Markham, Ontario, Canada, site investigator);

Susan Perlman, MD (UCLA Medical Center, Los Angeles, California, USA, site investigator);

Michael D. Geschwind, MD, PhD and Sharon Sha, MD (University of California, San Francisco, California, USA, site investigators);

Tom Warner, MD, PhD (National Hospital for Neurology and Neurosurgery, London, UK, site investigator);

Anne Rosser, MD, PhD, MRCP (Cardiff University, Cardiff, Wales, UK, site investigator);

Frederick Marshall, MD (University of Rochester, Rochester, New York, USA, site investigator);

Peter Panegyres, MB, BS, PhD, and Joseph Lee, MBBS, DPM, MCIL, MRCPsych, FHKCPsych, FHKAM (Psych), FRANZCP (Neurosciences Unit, Graylands, Selby-Lemnos & Special Care Health Services, Perth, Australia, site investigators);

Joel Perlmutter, MD (Washington University, St. Louis, Missouri, USA, site investigator);

Zosia Miedzybrodzka, MD, PhD, (Clinical Genetics Centre, Aberdeen, Scotland, UK, site investigator);

David Craufurd, MD (University of Manchester, Manchester, UK, site investigator);

Pietro Mazzoni, MD, PhD (site investigator), and Karen Marder, MD, PhD (administrative investigator) (Columbia University Medical Center, New York, New York, USA);

Rajeev Kumar, MD, (Colorado Neurological Institute, Englewood, Colorado, USA, site investigator);

Vicki Wheelock, MD (University of California, Davis, Sacramento, California, USA, site investigator);

Wayne Martin, MD, and Oksana Suchowersky, MD (University of Alberta, Edmonton, Alberta, Canada, site investigators);

Anwar Ahmed, MD (site investigator), and Stephen Rao, MD (administrative investigator) (Cleveland Clinic Foundation, Cleveland, Ohio, USA).

Executive Committee

Principal Investigator Jane S. Paulsen, PhD, Jeffrey D. Long, PhD, Hans J. Johnson, PhD, Thomas Brashers-Krug, MD, Phil Danzer, BBA, Amanda Miller, LMSW, H. Jeremy Bockholt, BS, and Kelsey Montross, BA (University of Iowa).

PREDICT-HD Contributors

Isabella De Soriano and Courtney Shadrick (University of Iowa, Iowa City, Iowa, USA, site coordinators);

Joy Preston (site coordinator), Anita Goh (site coordinator), and Stephanie Antonopoulos (cognitive rater) (St. Vincent’s Hospital, The University of Melbourne, Kew, Victoria, Australia);

Angela Komiti (The University of Melbourne, Royal Melbourne Hospital, Melbourne, Australia, site coordinator);

Joji Decolongon (site coordinator), Mannie Fan (site coordinator), and Allison Coleman (cognitive rater) (University of British Columbia, Vancouver, British Columbia, Canada);

Mark Varvaris (cognitive rater), Maryjane Ong (site coordinator), and Nadine Yoritomo (site coordinator) (Johns Hopkins University, Baltimore, Maryland, USA);

Greg Suter (Hereditary Neurological Disease Centre, Wichita, Kansas, USA, site coordinator);

Emily P. Freney and Alma Macaraeg (University of Washington and VA Puget Sound Health Care System, Seattle, Washington, USA, site coordinators);

Cathy Wood-Siverio (site coordinator) and Stewart A. Factor (motor rater) (Emory University School of Medicine, Atlanta, Georgia, USA);

Sarah Mason (site coordinator) and Natalie Valle Guzman (cognitive rater) (John van Geest Centre for Brain Repair, Cambridge, UK);

Jane Griffith, Jillian McMillan, and David Gunn (Westmead Hospital, Sydney, Australia, site coordinators);

Katrin Barth (site coordinator), Sonja Trautmann (site coordinator), Daniela Schwenk (cognitive rater), and Carolin Eschenbach (cognitive rater) (University of Ulm, Ulm, Germany);

Melissa Wesson (site coordinator) and Joanne Wojcieszek (motor rater) (Indiana University School of Medicine, Indianapolis, IN, USA);

Alanna Sheinberg (cognitive rater), Albie Law (site coordinator), and Irita Karmalkar (site coordinator) (Centre for Addiction and Mental Health, University of Toronto, Markham, Ontario, Canada);

Brian Clemente (UCLA Medical Center, Los Angeles, California, USA, site coordinator);

Joseph Winer and Gabriela Satris (University of California, San Francisco, California, USA, site coordinators);

Maggie Burrows (National Hospital for Neurology and Neurosurgery, London, UK, site coordinator);

Kathy Price and Sarah Hunt (Cardiff University, Cardiff, Wales, UK, site coordinators);

Amy Chesire (site coordinator), Mary Wodarski (cognitive rater), and Charlyne Hickey (motor rater) (University of Rochester, Rochester, New York, USA);

Maria Tedesco and Brenton Maxwell (Neurosciences Unit, Graylands, Selby-Lemnos & Special Care Health Services, Perth, Australia, site coordinators);

Stacey Barton and Shineeka Smith (Washington University, St. Louis, Missouri, USA, site coordinators);

Daniela Rae, Vivien Vaughan, and Mariella D’Alessandro (Clinical Genetics Centre, Aberdeen, Scotland, UK, site investigators);

Judith Bek (site coordinator) and Elizabeth Howard (motor rater) (University of Manchester, Manchester, UK);

Paula Wasserman (Columbia University Medical Center, New York, New York, USA, site coordinator);

Diane Erickson (site coordinator), Christina Reeves (site coordinator), and Breanna Nickels (cognitive rater) (Colorado Neurological Institute, Englewood, Colorado, USA);

Lisa Kjer (site coordinator), Amanda Martin (site coordinator), and Sarah Farias (cognitive rater) (University of California, Davis, Sacramento, California, USA);

Pamela King (site coordinator), Marguerite Wieler (site coordinator), and Satwinder Sran (cognitive rater) (University of Alberta, Edmonton, Alberta, Canada);

Christine Reece, Alex Bura, and Lyla Mourany (Cleveland Clinic Foundation, Cleveland, Ohio, USA, site coordinators).

# Supplementary Figures and Tables

**
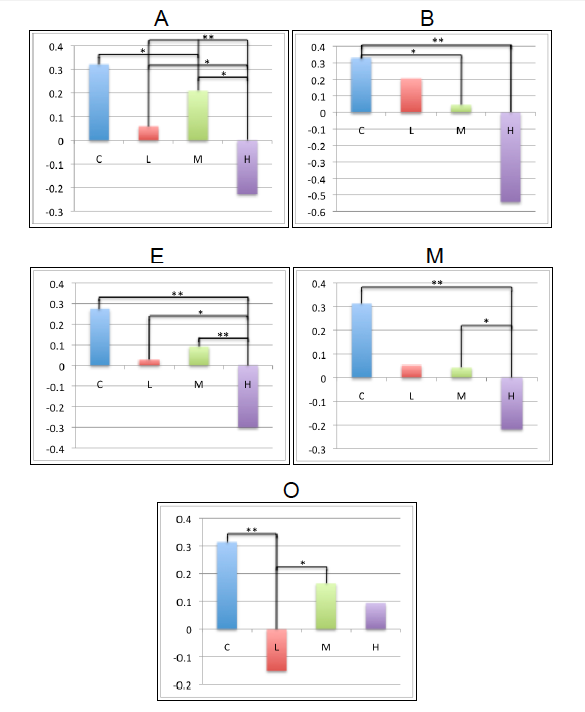
**

**Supplementary Figure 1.** Significant SBM Components in Supplemental CAPD-Group Contrasts. Significant SBM components in Low > Medium, Low > High, and Medium > High CAP-group contrasts (p<0.05) A-T. Comparisons labeled with a single asterisk (*) are significant at p <0.05; Two asterisks (**) denotes significance of p<0.001.


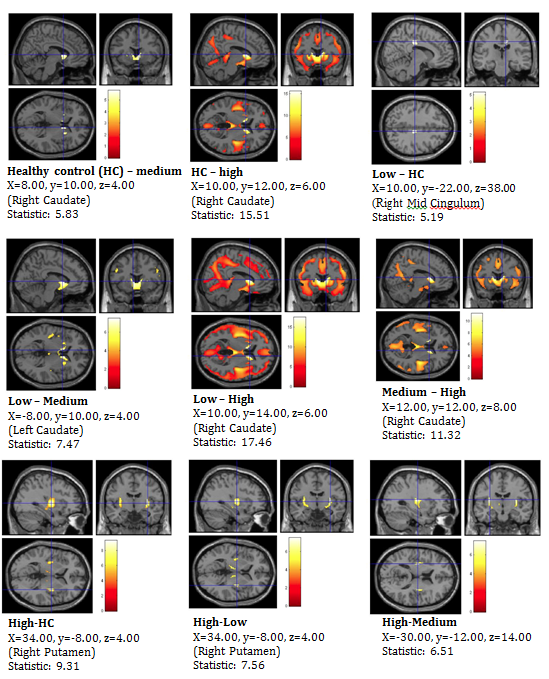


**Supplementary Figure 2**: VBM significant group effects (ANCOVA T contrasts). Pairwise effects of CAP group on VBM, showing regions that vary throughout the prodrome (p<0.05). The colored bar legend codes the T-statistic at each voxel, with white areas denoting voxels with the highest significance levels.

**
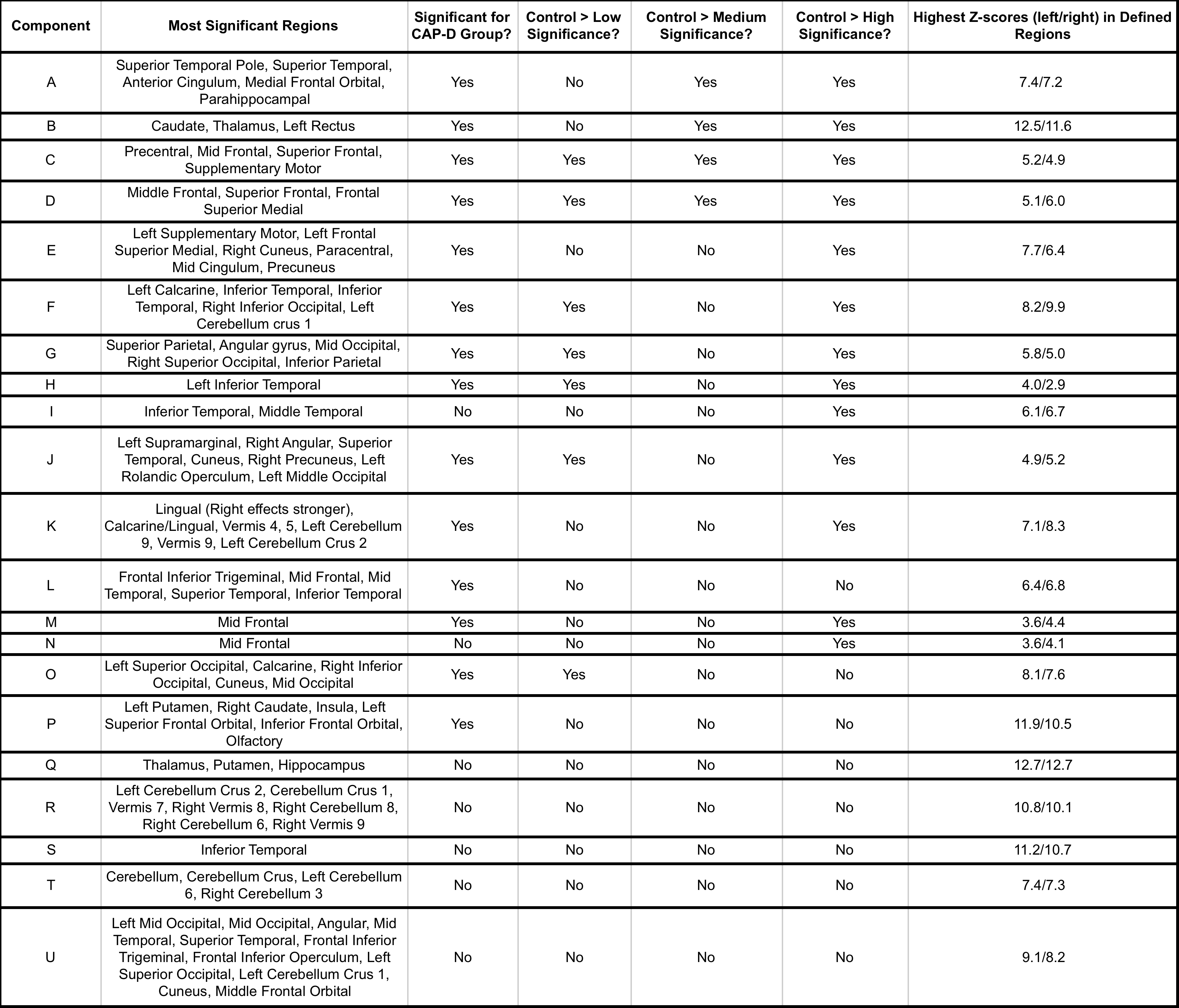
**

**Supplementary Table 1**: SBM Component Descriptions. Summary of each SBM component, including MANCOVA CAP-group significance, Control > Low, Control > Medium, and Control > High Sidak pairwise contrast significance, regions most substantially contributing to SBM components (Z-scores of at least 4.0), and left- and right- hemisphere maximum z scores.

**
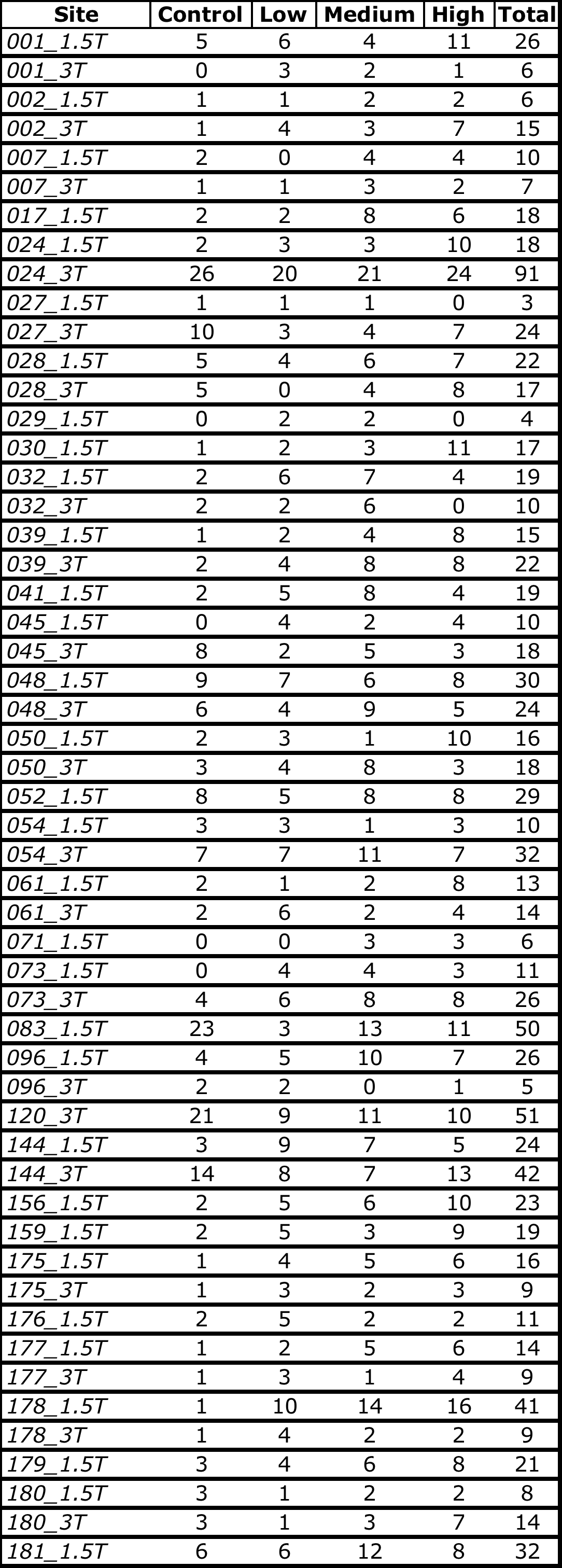
**

**Supplementary Table 2**: Site Demographics. Scan Site information, including site number and scanner field strength in tesla (T) (formatted in column one as site-number_field-strength). Also presented are the numbers of control and low-, medium-, and high-prodromal subjects at each scan site, and total participant numbers for each site.
